# Bezafibrate for X-Linked Adrenoleukodystrophy

**DOI:** 10.1371/journal.pone.0041013

**Published:** 2012-07-20

**Authors:** Marc Engelen, Luc Tran, Rob Ofman, Josephine Brennecke, Ann B. Moser, Inge M. E. Dijkstra, Ronald J. A. Wanders, Bwee Tien Poll-The, Stephan Kemp

**Affiliations:** 1 Department of Neurology, Academic Medical Center, University of Amsterdam, Amsterdam, The Netherlands; 2 Department of Clinical Chemistry, Laboratory Genetic Metabolic Diseases, Academic Medical Center, University of Amsterdam, Amsterdam, The Netherlands; 3 Department of Pediatric Neurology/Emma Children’s Hospital, Academic Medical Center, University of Amsterdam, Amsterdam, The Netherlands; 4 Department of Neurogenetics, Kennedy Krieger Institute, Baltimore, Maryland, United States of America; Hôpital Robert Debré, France

## Abstract

X-linked adrenoleukodystrophy (X-ALD) is caused by mutations in the *ABCD1* gene and is characterized by impaired beta-oxidation of very-long-chain fatty acids (VLCFA) and subsequent VLCFA accumulation in tissues. In adulthood X-ALD most commonly manifests as a gradually progressive myelopathy, (adrenomyeloneuropathy; AMN) without any curative or disease modifying treatments. We recently showed that bezafibrate (BF), a drug used for the treatment of hyperlipidaemia, reduces VLCFA accumulation in X-ALD fibroblasts by inhibiting ELOVL1, an enzyme involved in the VLCFA synthesis. We therefore designed a proof-of-principal clinical trial to determine whether BF reduces VLCFA levels in plasma and lymphocytes of X-ALD patients. Ten males with AMN were treated with BF for 12 weeks at a dose of 400 mg daily, followed by 12 weeks of 800 mg daily. Every 4 weeks patients were evaluated for side effects and blood samples were taken for analysis. Adherence was good as indicated by a clear reduction in triglycerides. There was no reduction in VLCFA in either plasma or lymphocytes. Plasma levels of BF did not exceed 25 µmol/L. We concluded that BF, at least in the dose given, is unable to lower VLCFA levels in plasma or lymphocytes in X-ALD patients. It is unclear whether this is due to the low levels of BF reached in plasma. Our future work is aimed at the identification of highly-specific inhibitors of ELOVL1 that act at much lower concentrations than BF and are well tolerated. BF appears to have no therapeutic utility in X-ALD.

**Trial Registration:**

ClinicalTrials.gov NCT01165060

## Introduction

X-linked adrenoleukodystrophy (X-ALD) is a peroxisomal disorder characterized by impaired β-oxidation of very long-chain fatty acids (VLCFA) and accumulation of these VLCFA in tissues [1]. It is caused by mutations in the *ABCD1* gene (www.x-ald.nl) [2]. The disease is highly variable in clinical expression, however, in adulthood it most frequently manifests as a gradually progressive myelopathy and peripheral neuropathy (adrenomyeloneuropathy phenotype or AMN) [1]. Treatment for AMN is purely symptomatic and currently there is no proven intervention that can halt progression of the disease [1]. We identified ELOVL1 as the enzyme responsible for the synthesis of VLCFA [3], and demonstrated that siRNA-mediated knockdown of ELOVL1 lowers VLCFA levels in X-ALD fibroblasts [3]. Next, we showed that bezafibrate (BF) reduces VLCFA levels in X-ALD fibroblasts by directly inhibiting ELOVL1 [4].

BF is a drug of the fibrate class for the treatment of dyslipidaemia and has a proven safety profile for (long-term) use in humans [5]. We therefore designed a proof of principal clinical trial to test whether BF can reduce VLCFA levels in the plasma and lymphocytes of patients with X-ALD.

## Methods

**Figure 1:**
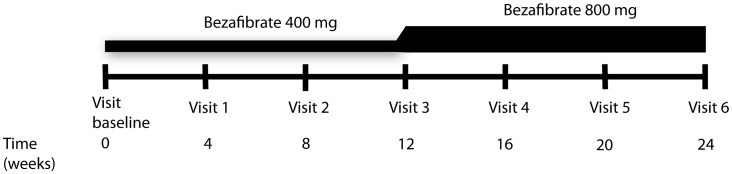
Schematic representation of the BEZA trial design.

The protocol for this trial and supporting CONSORT checklist are available as supporting information; see Checklist S1 and Protocol S1. The BEZA trial study protocol was approved by the Institutional Review Board (Medisch Ethische Toetsings Commissie) of the Academic Medical Center. The trial is registered at clinicaltrials.gov (NCT01165060). Adult men with biochemically and genetically proven X-ALD without contra-indications for the use of BF were eligible for inclusion. All participating patients were evaluated at baseline for eligibility and received trial medication after written informed consent was obtained. They were evaluated at intervals of 4 weeks until the end of the trial at 24 weeks. The initial dose of BF was 400 mg per day, which was subsequently increased to 800 mg per day at week 12 (Figure 1). At each visit side effects were monitored, a general physical examination including weight was performed and blood samples taken. Blood samples were taken in the morning after an overnight fast before the first medication dose. Blood samples were analyzed at the laboratory for clinical chemistry for routine laboratory tests. VLCFA and BF levels were analyzed as previously described [6], [7]. Lysophosphatidylcholine-C26∶0 (C26∶0 lysoPC) was analyzed in bloodspots [8]. Data were analyzed with PASW statistics, version 18 (IBM). Statistical significance was evaluated with a paired t-test.

## Results

Ten males with AMN participated in the trial. No side effects that necessitated discontinuation of the trial medication occurred. Body weight was unchanged (Table 1). There was a clear reduction in plasma triglycerides (1.34 mmol/L to 0.70 mmol/L at BF 400 mg and 0.71 mmol/L at BF 800 mg), and to a lesser extent a decrease in total cholesterol and LDL-cholesterol. There was also an increase in HDL-cholesterol (Table 1). These are known effects of BF and confirm patient adherence. There was no consistent reduction in C26∶0 in plasma or lymphocytes, neither at 400 nor at 800 mg BF per day (Table 1). We observed an increase in plasma C22∶0 and C24∶0 at a dose of 800 mg BF per day. The amount of C26∶0 lysoPC was unchanged in blood spots after 24 weeks of treatment with BF. The plasma level of BF did not exceed 25 µmol/L at the highest dose of 800 mg BF per day.

**Table 1:** Summary of the different parameters measured at the indicated time point in the trial.

Plasma	Baseline	BF 400 mg	BF 800 mg
Total cholesterol (mmol/L)	5.57±1.42	4.80±0.88**	4.85±0.84*
LDL(mmol/L)	3.67±1.17	2.93±0.80**	2.90±0.73*
HDL(mmol/L)	1.39±0.25	1.57±0.25***	1.64±0.26***
TG(mmol/L)	1.34±0.79	0.70±0.31**	0.71±0.22*
C22∶0(µmol/L)	43.32±9.25	45.37±8.12	55.02±9.74**
C24∶0(µmol/L)	62.56±11.68	64.08±11.78	82.62±12.50***
C26∶0(µmol/L)	3.26±0.96	2.56±0.50**	2.99±0.49
C26∶0/C22∶0 ratio	0.075±0.015	0.058±0.012	0.056±0.012**
Bezafibrate(µmol/L)	n.d.	n.d.	10.1±6.7
**Lymphocytes**			
C22∶0(nmol/mg)	5.89±1.03	5.85±1.65	5.10±1.47
C24∶0(nmol/mg)	6.03±0.78	6.45±1.85	6.42±1.44
C26∶0(nmol/mg)	0.35±0.040	0.37±0.084	0.40±0.11
C26∶0/C22∶0 ratio	0.06±0.01	0.06±0.01	0.08±0.03
**bloodspots**			
C26∶0 lysoPC	2.84±1.40	2.50±0.89	2.63±1.18
Weight (kg)	86.7±9.3	n.d.	87.9±10.5

Values are mean ± the standard deviation. Statistically significant differences from the baseline value are indicated.

* p<0.05,

** p<0.01,

*** p<0.001. n.d. = not determined.

## Discussion

The pathophysiology of X-ALD is not well understood, although it seems likely that accumulation of VLCFA is toxic and related to neurodegeneration [9]. Therefore drugs that reduce the level of VLCFA might be effective in halting or slowing progression of the disease.

Recently, we showed that it is possible to reduce VLCFA in fibroblasts from X-ALD patients by inhibiting the synthesis of VLCFA by the enzyme ELOVL1 [3]. We later showed that this can also be accomplished by incubating fibroblasts from X-ALD patients with BF [4].

BF is a drug that has been in use for decades for the treatment of hypertriglyceridaemia and has an excellent safety profile [5]. Therefore we decided to initiate this small scale proof of principle clinical trial to investigate whether BF reduces VLCFA in plasma and lymphocytes of X-ALD patients. In a previous clinical trial with lovastatin we demonstrated that reduction of plasma VLCFA can be an artifact of LDL reduction and does not reflect a reduction in blood cells [10].

Unfortunately, we could not show a reduction on plasma or lymphocyte VLCFA levels. Conversely, there was an unexpected increase in C22∶0 and C24∶0 levels in plasma. We did not observe this in blood cells or bloodspots.

Our results show that there is no rationale for a large follow-up trial with clinical endpoints utilizing this compound.

The concept of treating X-ALD patients with an inhibitor of VLCFA synthesis remains a feasible option. It seems that BF is simply not efficacious enough. Our previous work suggests that BF is a competitive inhibitor of ELOVL1 [4]. In our cell culture experiments a high concentration of BF of 400 µmol/L was required to achieve a maximal effect on the level of VLCFA. At this concentration the *de novo* VLCFA synthesis was reduced to the level in control cells. It is likely that even with the high dose of 800 mg BF per day, the intracellular levels of BF remained inadequate. Indeed, at the highest BF dosage plasma levels did not exceed 25 µmol/L with an average of 10 µmol/L (Table 1). These levels are not peak levels, but rather residual plasma levels. It is unlikely that concentrations even approaching 400 µmol/L were reached. This may explain the lack of *in vivo* efficacy of BF on our outcome parameters. To achieve the effect of VLCFA reduction, significantly higher BF concentrations are necessary as compared to concentrations indicated for reduction of TG.

Future research will be focused on the identification of specific inhibitors of ELOVL1 that act at much lower concentrations than BF and are well-tolerated. In conclusion, BF appears to have no therapeutic utility in X-ALD.

## Supporting Information

Checklist S1 **CONSORT Checklist.**
(DOC)

Protocol S1 **Trial Protocol.**
(PDF)
