# Supplementary material for: Bezafibrate for X-Linked Adrenoleukodystrophy
Source: PLoS One. 2012 Jul 20;7(7):e41013. doi: 10.1371/journal.pone.0041013 (PMC3401223; doi:10.1371/journal.pone.0041013)

# Effect of bezafibrate on very long chain fatty acid metabolism in men with X-linked adrenoleukodystrophy (X-ALD)

Drs. Marc Engelen, AIOS neurologie, Afdeling Neurologie, AMC
Dr. Stephan Kemp, Moleculair bioloog, Laboratorium voor Genetische Metabole Ziekten, AMC
Prof. Dr. Bwee-Tien Poll – The, Kinderneuroloog, Afdeling Kinderneurologie, AMC

## Effect of bezafibrate on very long chain fatty acid metabolism in men with X-linked adrenoleukodystrophy (X-ALD)

| | |
|---|---|
| **Protocol ID** | **Prot_ME_2009_0819**<br>**MEC 09/278** |
| **Short title** | **BEZA trial** |
| **Version** | **3.0** |
| **Date** | **23-04-2009** |
| **Coordinating investigator/project leader** | ***Prof. Dr. Poll – The***<br>***H8-240***<br>***b.t.pollthe@amc.uva.nl*** |
| **Principal investigator(s) (in Dutch: hoofdonderzoeker/uitvoerder)**<br>***Multicenter research: per site*** | ***Prof. Dr. Poll – The***<br>***H8-240***<br>***b.t.pollthe@amc.uva.nl*** |
| **Sponsor (in Dutch: verrichter/opdrachtgever)** | ***Academisch Medisch Centrum*** |
| **Independent physician(s)** | **Dr. G.J.M. Walstra**<br>**H2-231**<br>**g.j.walstra@amc.uva.nl** |
| **Laboratory sites** | ***Laboratorium voor Genetische Metabole Ziekten***<br>**F0-225**<br>**65958** |
| **Pharmacy** | ***Ziekenhuisapotheek AMC*** |

**PROTOCOL SIGNATURE SHEET**

| Name | Signature | Date |
|---|---|---|
| **Coordinating Investigator/Project leader/Principal Investigator:**<br>***Prof. Dr. B.T. Poll - The*** | | |
| | | |

## TABLE OF CONTENTS

1. INTRODUCTION AND RATIONALE ..... 8
2. OBJECTIVES ..... 10
3. STUDY DESIGN ..... 11
4. STUDY POPULATION ..... 12
4.1 Population (base) ..... 12
4.2 Inclusion criteria ..... 12
4.3 Exclusion criteria ..... 12
4.4 Sample size calculation ..... 12
5. TREATMENT OF SUBJECTS ..... 13
5.1 Investigational product/treatment ..... 13
5.2 Use of co-intervention (if applicable) ..... 13
5.3 Escape medication (if applicable) ..... 13
6. INVESTIGATIONAL MEDICINAL PRODUCT ..... 14
6.1 Name and description of investigational medicinal product ..... 14
6.2 Summary of findings from non-clinical studies ..... 14
6.3 Summary of findings from clinical studies ..... 14
6.4 Summary of known and potential risks and benefits ..... 14
6.5 Description and justification of route of administration and dosage ..... 14
6.6 Dosages, dosage modifications and method of administration ..... 14
6.7 Preparation and labelling of Investigational Medicinal Product ..... 15
6.8 Drug accountability ..... 15
7. METHODS ..... 16
7.1 Study parameters/endpoints ..... 16
7.1.1 Main study parameter/endpoint ..... 16
7.1.2 Secondary study parameters/endpoints (if applicable) ..... 16
7.1.3 Other study parameters (if applicable) ..... 16
7.2 Randomisation, blinding and treatment allocation ..... 16
7.3 Study procedures ..... 16
7.4 Withdrawal of individual subjects ..... 16
7.4.1 Specific criteria for withdrawal (if applicable) ..... 16
7.5 Replacement of individual subjects after withdrawal ..... 17
7.6 Follow-up of subjects withdrawn from treatment ..... 17
7.7 Premature termination of the study ..... 17
8. SAFETY REPORTING ..... 18
8.1 Section 10 WMO event ..... 18
8.2 Adverse and serious adverse events ..... 18
8.2.1 Suspected unexpected serious adverse reactions (SUSAR) ..... 19
8.2.2 Annual safety report ..... 19
8.3 Follow-up of adverse events ..... 20
8.4 Data Safety Monitoring Board (DSMB) ..... 20
9. STATISTICAL ANALYSIS ..... 21

9.1 Descriptive statistics ........ 21
9.2 Univariate analysis ........ 21
9.3 Multivariate analysis ........ 21
9.4 Interim analysis (if applicable) ........ 21
10. ETHICAL CONSIDERATIONS ........ 22
10.1 Regulation statement ........ 22
10.2 Recruitment and consent ........ 22
10.3 Objection by minors or incapacitated subjects (if applicable) ........ 22
10.4 Benefits and risks assessment, group relatedness ........ 22
10.5 Compensation for injury ........ 22
10.6 Incentives (if applicable) ........ 23
11. ADMINISTRATIVE ASPECTS AND PUBLICATION ........ 24
11.1 Handling and storage of data and documents ........ 24
11.2 Amendments ........ 24
11.3 Annual progress report ........ 24
11.4 End of study report ........ 24
11.5 Public disclosure and publication policy ........ 25
12. REFERENCES ........ 26
13 TABLES AND FIGURES ........ 27

## LIST OF ABBREVIATIONS AND RELEVANT DEFINITIONS

| | |
|---|---|
| ABR | ABR form (General Assessment and Registration form) is the application form that is required for submission to the accredited Ethics Committee (ABR = Algemene Beoordeling en Registratie) |
| AE | Adverse Event |
| AR | Adverse Reaction |
| CA | Competent Authority |
| CCMO | Central Committee on Research Involving Human Subjects |
| CV | Curriculum Vitae |
| DSMB | Data Safety Monitoring Board |
| EU | European Union |
| EudraCT | European drug regulatory affairs Clinical Trials GCP Good Clinical Practice |
| IB | Investigator's Brochure |
| IC | Informed Consent |
| IMP | Investigational Medicinal Product |
| IMPD | Investigational Medicinal Product Dossier |
| METC | Medical research ethics committee (MREC); in Dutch: medisch ethische toetsing commissie (METC) |
| (S)AE | Serious Adverse Event |
| SPC | Summary of Product Characteristics (in Dutch: officiële productinfomatie IB1-tekst) |
| Sponsor | The sponsor is the party that commissions the organisation or performance of the research, for example a pharmaceutical company, academic hospital, scientific organisation or investigator. A party that provides funding for a study but does not commission it is not regarded as the sponsor, but referred to as a subsidising party. |
| SUSAR | Suspected Unexpected Serious Adverse Reaction |
| Wbp | Personal Data Protection Act (in Dutch: Wet Bescherming Persoonsgevens) |
| WMO | Medical Research Involving Human Subjects Act (Wet Medisch-wetenschappelijk Onderzoek met Mensen |

## SUMMARY

**Rationale:** X-linked adrenoleukodystrophy (X-ALD) is a peroxisomal disorder, characterized by accumulation of very long-chain fatty acids (VLCFA; >C22:0) with highly variable expression. For a large category of patients there is no curative treatment. Experiments with bezafibrate in primary fibroblasts from patients with X-ALD and X-ALD knockout mice suggest bezafibrate can reduce VLCFA accumulation in X-ALD, the biochemical hallmark of the disease. This warrants further study to see if these findings can be reproduced in patients with X-ALD.

**Objective**: To determine if bezafibrate can reduce VLCFA in plasma, leukocytes and erythrocytes from patients with X-ALD.

**Study design:** Open-label intervention study with biochemical outcome parameters.

**Study population:** Adult patients with confirmed X-ALD (adrenomyeloneuropathy) without contra-indications for the use of bezafibrate are eligible to participate.

**Intervention (if applicable)**: Participating patients will receive Bezalip Retard 400 mg for three months, followed by 800 mg during three months.

**Main study parameters/endpoints:** Primary endpoints are cholesterol (total-, LDL-, and HDL-cholesterol) and triglycerides in plasma, VLCFA (C22:0, C24:0 and C26:0) in plasma, leukocytes and erythrocytes, and C26:0-lysophosphatidylcholine in blood. Secondary outcomes are side effects reported by patients and abnormalities in the safety lab.

**Nature and extent of the burden and risks associated with participation, benefit and group relatedness:** The risk of participation is estimated to be low. Bezafibrate is only rarely associated with (reversible) severe adverse reactions (in < 0.01% rhabdomyolysis, thrombocytopenia or leukopenia have been reported).

# 1. INTRODUCTION AND RATIONALE

*X-linked adrenoleukodystrophy (X-ALD)*

X-linked adrenoleukodystrophy is an inherited metabolic disorder characterised by defective β-oxidation of very long chain fatty acids (fatty acids with a carbon backbone ≥ 22). [1] The disease is rare: the estimated birth incidence is 1 in 17.000 newborns. [2] The clinical phenotype of the disorder is highly variable. [1] Knowledge of incidence and pathophysiology of X-ALD and peroxisomal disorders in general has increased significantly over the last few decades.

*Symptoms and disease progression*

The expression and severity of the disease show a large variability, also within the same kindred with identical *ABCD1* mutations and similar environmental influences. [2] This phenotypic variability remains largely unexplained.

Clinically, the disease can be classified into several phenotypes. [1] However, phenotypes can progress to other phenotypes over time. [3] The most frequent phenotype is adrenomyeloneuropathy (AMN). This phenotype becomes manifest in early adulthood (usually in the 3rd decade) and is mainly characterised by myelopathy and peripheral neuropathy with incontinence and spastic paraparesis. The symptoms are gradually progressive and eventually cause significant impairment in the activities of daily living.

X-ALD can also cause severe and rapidly progressive cerebral demyelination in childhood, with the highest incidence between 3 and 10 years of age. [1] This is the Childhood Cerebral ALD (CCALD) phenotype and if not treated by bone marrow transplantion early in the course of the disease is relentlessly progressive and fatal. [4]

*Pathophysiology*

X-ALD is caused by mutations in the *ABCD1* gene coding for adrenoleukodystrophy protein (ALDP). [1] ALDP is located in the peroxisomal membrane and functions as a transmembrane transport protein involved in transporting VLCFA into the peroxisomal matrix. VLCFA are exclusively degraded by beta-oxidation in the peroxisome. Dysfunction of deficiency of ALDP causes defective beta-oxidation and accumulation of VLCFA. [5] How this VLCFA accumulation causes the symptoms of the disease (e.g. direct toxicity or other indirect mechanisms) remains unclear, but destabilizing effects of VLCFA on the cell membrane has been demonstrated. [6] In the cerebral white matter of patients affected by CCALD there is severe inflammation, but the trigger remains unknown. To date many different *ABCD1* mutations have been described (http://www.x-ald.nl), but genotype-phenotype correlation has never been established. [7]

*Therapy*

To date, there is a treatment for early stage CCALD. Allogenic bone marrow transplantation is effective in halting and even reversing the disease process in this category of patients. [4] This treatment still carries significant morbidity and mortality. Long term follow up is indicates these patients are at risk to develop AMN (Aubourg, unpublished data). The 4:1 mixture of glyceryl trioleate and glyceryl trierucate ("Lorenzo's Oil") that is still being prescribed to some

patient with X-ALD does reduce plasma VLCFA to normal levels within weeks, but does not seem to have an effect on disease progression. [8, 9] Lovastatin was reported to reduce VLCFA, [10] but a more recent study proves this is not a real reduction and it should not be considered a treatment for X-ALD (M. Engelen et al, NEJM, in press). The effect on VLCFA reached a steady state after 3 months of treatment (M. Engelen et al, NEJM, in press).

For the other phenotypes of X-ALD only symptomatic treatment is available, such as hormone replacement therapy for adrenal insufficiency or treatment of spasticity.

*Rationale for the use of bezafibrate in X-ALD*

Recent experiments in cultured fibroblasts from patients with X-ALD show that incubation with bezafibrate can reduce VLCFA levels in these cells by more than 40% with no apparent toxicity (M. Engelen and S. Kemp, manuscript in preparation). It is remarkable that this effect does not seem to be caused by increased VLCFA degradation, but rather by inhibiting the synthesis of VLCFA. This is in accordance with previous work on bezafibrate (and several other drugs from the same class), suggesting that this drug can inhibit fatty acid synthesis. [11, 12] Follow-up experiments in X-ALD knockout mice showed a reduction of VLCFA accumulation the liver of these animals after 8 weeks of treatment with bezafibrate (administered in the chow). In the plasma of the treated animals we could also detect a decrease in C26:0-lysophosphatidylcholine, a marker for VLCFA accumulation (Hubbard et al., Mol Genet Metab 2006).

These observations provide a rationale for the use of bezafibrate as a possible target compound for the reduction of VLCFA in patients with X-ALD, and therefore a potential new treatment for this disorder that might affect progression of the disease.

Bezafibrate is an approved drug in the Netherlands, and is registered to reduce plasma VLDL- and LDL-lipoprotein concentration (and therefore reduces plasma cholesterol and triglycerides). Currently, it is used for the treatment of hypertriglyceridaemia. [13] This drug has been in use for several decades and is generally considered safe.

The current trial is designed as a “proof of principle” trial. Our aim is to determine if our *in vitro* data and *in vivo* data from mice can be extrapolated to patients with X-ALD. Therefore, we designed a small trial to see if there is indeed a biochemical effect (e.g. reduction of VLCFA). If there is indeed an effect on VLCFA a new large-scale placebo controlled trial with long term follow up and clinical outcome parameters will be necessary.

## 2. OBJECTIVES

Primary Objective: The primary objective of this study is to determine if bezafibrate can reduce VLCFA in plasma, lymphocytes and erythrocytes and C26:0-lysophosphatidylcholine in patients with X-linked adrenoleukodystrophy.

## 3. STUDY DESIGN

The study is a "proof of principle" pilot study with only biochemical outcome parameters. It is an open-label intervention study. After inclusion and drawing of a blood sample for determining baseline values of the outcome parameters, patients will start with trial medication. There is a dose escalation; patients will use a standard dose of 400 mg daily, which will be increased by 800 mg after 3 months, to a maximum of 800 mg. This will help to determine the most effective dosage for a possible follow-up trial. A flow chart (Figure 1) and Table 1 illustrate the design of the trial.

# 4. STUDY POPULATION

## 4.1 Population (base)

The birth incidence of X-linked adrenoleukodystrophy is 1 in 17000 live births (this includes males and female hemizygotes). The AMC is the referral centre for this disorder, and consequently most of the Dutch population visits the AMC for follow-up, about 150 come in for regular check-ups. For this study, we will consider adult male patients with the adrenomyeloneuropathy (AMN) phenotype of X-ALD. There is a patient organisation (Belangenvereniging X-ALD, http://www.xald.nl) with a regular newsletter that can help reach patients that do not visit the hospital for follow-up.

## 4.2 Inclusion criteria

Inclusion criteria for participation in this trial are:

- an age of 18 years or older
- capable of giving informed consent and physically capable of visiting the hospital for follow-up visits
- no contra-indications for the use of bezafibrate, e.g. kidney- and/or liver disease.
- confirmed X-ALD, AMN phenotype (confirmed by VLCFA analysis and mutation analysis of the *ABCD1* gene)

## 4.3 Exclusion criteria

Exclusion criteria for participation in this trial are:

- use of medication that lowers cholesterol and/or triglycerides (e.g. statins)
- liver disease or and increase in serum CK of more than 3 times the baseline level
- treatment with Lorenzo's oil in the 8 weeks preceding the trial

## 4.4 Sample size calculation

If 8 patients are enrolled in the trial, a 50% reduction in plasma C26:0 can be detected with a power of 80%, using tandem-MS analysis to determine plasma C26:0 levels.

## 5. TREATMENT OF SUBJECTS

### 5.1 Investigational product/treatment

All patients will be treated with bezafibrate (up to 800 mg once daily). Bezafibrate is a drug used for the treatment of hypertriglyceridaemia. For this trial Bezalip Retard tablets will be used, that can be taken once daily. Contra-indications for the use of this medication are liver- and/or kidney disorders. Common side effects include dyspepsia and dizziness. Rare side-effects (< 0.01%) are rhabdomyolysis, thrombocytopenia or leukopenia.

### 5.2 Use of co-intervention (if applicable)

A co-intervention has not been planned.

### 5.3 Escape medication (if applicable)

There is no escape medication available. If side-effects are reported or the safety lab done at each visit is abnormal, the medication will be discontinued.

## 6. INVESTIGATIONAL MEDICINAL PRODUCT

For more detailed information please refer to the SPC for bezafibrate (Bezalip) included with this protocol.

### 6.1 Name and description of investigational medicinal product

Bezafibrate (Bezalip) Retard 400 mg.

### 6.2 Summary of findings from non-clinical studies

For more detailed information please refer to the SPC for bezafibrate (Bezalip) included with this protocol, more specifically page 7.

### 6.3 Summary of findings from clinical studies

Bezafibrate is used to reduce cholesterol and/or triglycerides in patients with hypertriglyceridaemia. There are no trials documenting the effect of bezafibrate on the levels of VLCFA in plasma in patients with X-ALD. The rationale is detailed in the introduction of this protocol.

### 6.4 Summary of known and potential risks and benefits

Several serious adverse reactions to bezafibrate have been described (rhabdomyolysis, thrombocytopenia and leukopenia) to occur in rare cases (< 0.01%). Pathophysiologically, it is not likely that patients with X-ALD are at greater risk for complications of bezafibrate than the general population. Benefits are unclear. If in this “proof of principle” study we can show that bezafibrate can indeed decrease VLCFA in plasma, leucocytes, and erythrocytes, there will be a rationale for a large clinical trial to determine if there is also clinical benefit for patients with this disorder.

### 6.5 Description and justification of route of administration and dosage

Bezafibrate can be administered orally. There is a sustained release tablet available (Bezalip Retard) that can be dosed once daily which is more convenient and is probably associated with better compliance than the non-sustained released tablets. In this trial the initial dose is 400 mg Bezalip retard once daily (the standard recommended dose), which will be increased in the second part of the trial to 800 mg once daily to establish if there is a dose-effect relationship.

### 6.6 Dosages, dosage modifications and method of administration

Bezafibrate will be administered as Bezalip Retard 400 mg once daily for 3 months, then the dosage will be increased to 400 mg twice daily for an additional 3 months. All trial medication will be taken orally.

## 6.7 Preparation and labelling of Investigational Medicinal Product

Medication will be ordered by the hospital pharmacy from Actavis, the manufacturer of Bezalip and labelled according to GCP requirements.

## 6.8 Drug accountability

Drug accountability will be handled by the hospital pharmacy of the AMC. Medication will be ordered though the hospital pharmacy, as well as drug delivery to the researchers for distribution among participating patients.

# 7. METHODS

## 7.1 Study parameters/endpoints

### 7.1.1 Main study parameter/endpoint

Primary endpoints are cholesterol (total-, LDL-, and HDL-cholesterol) and triglycerides in plasma, VLCFA (C22:0, C24:0 and C26:0) in plasma, leukocytes and erythrocytes, and C26:0-lysophosphatidylcholine in blood.

### 7.1.2 Secondary study parameters/endpoints (if applicable)

Secondary outcomes are side effects reported by patients and abnormalities in the safety lab.

### 7.1.3. Other study parameters (if applicable)

Other parameters are drop-out and compliance (as measured by an effect on plasma lipids).

## 7.2 Randomisation, blinding and treatment allocation

In this open-label pilot study there is no randomisation procedure.

## 7.3 Study procedures

At each of the visits a venapuncture will be performed for several tests (detailed in Table 1). All routine tests will be performed at the clinical chemistry department of the AMC. Measurement of VLCFA and C26:0-lysophosphatidylcholine (C26:0-lysophosphatidylcholine levels will be determined by A. Moser at the Kennedy Krieger Institute in Baltimore) will be performed at the Laboratorium voor Genetische Metabole Ziekten of the AMC. These venapuncutures are not part of routine treatment and are specifically for this trial.

## 7.4 Withdrawal of individual subjects

Subjects can leave the study at any time for any reason if they wish to do so without any consequences. The investigator can decide to withdraw a subject from the study for urgent medical reasons.

### 7.4.1 Specific criteria for withdrawal (if applicable)

- Abnormalities in the safety lab:
1. thrombocytopenia (thrombocytes < 50 * $10^9$ / L)
2. leukopenia (leukocytes < 4 * $10^9$ / L)
3. increase in ALAT or ASAT of more than 3 times the upper limit of normal.
4. increase in CK of more than 5 times the upper limit of normal
5. increase in creatinin to more than 30% of the baseline value

- Adverse events requiring medical care or hospitalisation that are probably related to the use of the trial medication.

### 7.5 Replacement of individual subjects after withdrawal

If possible, new patients will be recruited to replace those who decide to discontinue the trial or have to be withdrawn from the trial because of side-effects.

### 7.6 Follow-up of subjects withdrawn from treatment

Patients withdrawn from the study will receive standard follow-up at our X-ALD outpatient clinic. Data from the last available measurement will be used in the analysis.

### 7.7 Premature termination of the study

The investigators will consider discontinuation of the trial in the case of an SAE, or if more than 30% of patients have to be withdrawn form the study because of side-effects from the trial medication.

# 8. SAFETY REPORTING

## 8.1 Section 10 WMO event

In accordance to section 10, subsection 1, of the WMO, the investigator will inform the subjects and the reviewing accredited METC if anything occurs, on the basis of which it appears that the disadvantages of participation may be significantly greater than was foreseen in the research proposal. The study will be suspended pending further review by the accredited METC, except insofar as suspension would jeopardise the subjects' health. The investigator will take care that all subjects are kept informed.

## 8.2 Adverse and serious adverse events

At each visit patients will fill in questionnaires relating to possible side effects, and undergo a physical examination. Furthermore, safety lab will be examined by the trial physician the day of the visit. If side-effects are suspected on clinical grounds or on the basis of safety lab, trial medication will be discontinued and the patient treated if necessary. In the case of a SAE (as described below) this will be reported to the relevant authorities and discontinuation of the trial will be considered. If more than 30% of patients develop adverse events requiring discontinuation the trial will be discontinued.

Adverse events are defined as any undesirable experience occurring to a subject during a clinical trial, whether or not considered related to the investigational drug. All adverse events reported spontaneously by the subject or observed by the investigator or his staff will be recorded.

A serious adverse event is any untoward medical occurrence or effect that at any dose results in death;

- is life threatening (at the time of the event);
- requires hospitalisation or prolongation of existing inpatients' hospitalisation;
- results in persistent or significant disability or incapacity;
- is a congenital anomaly or birth defect;
- is a new event of the trial likely to affect the safety of the subjects, such as an unexpected outcome of an adverse reaction, lack of efficacy of an IMP used for the treatment of a life threatening disease, major safety finding from a newly completed animal study, etc.

All SAEs will be reported to the accredited METC that approved the protocol, according to the requirements of that METC.

### 8.2.1 Suspected unexpected serious adverse reactions (SUSAR)

Adverse reactions are all untoward and unintended responses to an investigational product related to any dose administered.

Unexpected adverse reactions are adverse reactions, of which the nature, or severity, is not consistent with the applicable product information (e.g. Investigator's Brochure for an unapproved IMP or Summary of Product Characteristics (SPC) for an authorised medicinal product).

The sponsor will report expedited the following SUSARs to the METC:

- SUSARs that have arisen in the clinical trial that was assessed by the METC;
- SUSARs that have arisen in other clinical trial of the same sponsor and with the same medicinal product, and that could have consequences for the safety of the subjects involved in the clinical trial that was assessed by the METC.

The remaining SUSARs are recorded in an overview list (line-listing) that will be submitted once every half year to the METC. This line-listing provides an overview of all SUSARs from the study medicine, accompanied by a brief report highlighting the main points of concern. The sponsor will report expedited all SUSARs to the competent authority, the Medicine Evaluation Board and the competent authorities in other Member States.

The expedited reporting will occur not later than 15 days after the sponsor has first knowledge of the adverse reactions. For fatal or life threatening cases the term will be maximal 7 days for a preliminary report with another 8 days for completion of the report.

### 8.2.2 Annual safety report

In addition to the expedited reporting of SUSARs, the sponsor will submit, once a year throughout the clinical trial, a safety report to the accredited METC, competent authority, Medicine Evaluation Board and competent authorities of the concerned Member States. This safety report consists of:

- a list of all suspected (unexpected or expected) serious adverse reactions, along with an aggregated summary table of all reported serious adverse reactions, ordered by organ system, per study;
- a report concerning the safety of the subjects, consisting of a complete safety analysis and an evaluation of the balance between the efficacy and the harmfulness of the medicine under investigation.

### 8.3 Follow-up of adverse events

All adverse events will be followed until they have abated, or until a stable situation has been reached. Depending on the event, follow up may require additional tests or medical procedures as indicated, and/or referral to the general physician or a medical specialist.

### 8.4 Data Safety Monitoring Board (DSMB)

An individual DSMB will not be established, considering the small scale and low risk of this trial. As described, the investigator will monitor participating patients and withdraw them from the study according to criteria described in 3.12.1.

# 9. STATISTICAL ANALYSIS

## 9.1 Descriptive statistics

The primary outcome data are continuous variables. Values of these variables will be compared with baseline pre-treatment values, and values obtained during treatment with 400 mg and 800 mg Bezalip. Analysis will be with a two-sided unpaired Student’s t-test.

## 9.2 Univariate analysis

Not applicable.

## 9.3 Multivariate analysis

Not applicable.

## 9.4 Interim analysis (if applicable)

There is no interim analysis planned of the primary outcome variables.

# 10. ETHICAL CONSIDERATIONS

## 10.1 Regulation statement

The study will be conducted according to the principles of the Declaration of Helsinki (http://www.wma.net/en/30publications/10policies/b3/index.html) and in accordance with the Medical Research Involving Human Subjects Act (WMO).

## 10.2 Recruitment and consent

Patient will be recruited through our outpatient clinic and the patient advocacy group (Belangenvereniging X-ALD, http://www.xald.nl). They will receive information about the trial both orally and in written form. The source of information might be an investigator and/or treating physician, since physicians in the X-ALD outpatient clinic usually are involved in patient care as well as research. Patients have no time-limit in deciding whether they want to participate in the trial. They can also contact an independent physician (Dr. G. Walstra, neurologist) to discuss any questions they might have.

## 10.3 Objection by minors or incapacitated subjects (if applicable)

Minors or adults not able to give informed consent (e.g. mental retardation) are not eligible for participation.

## 10.4 Benefits and risks assessment, group relatedness

As detailed previously, the estimated risk of participation in this trial for patients with X-ALD is small. Benefit is unclear. However, based on our observations *in vitro* and a study in X-ALD knockout mice there is a clear rationale for the study. If bezafibrate also has a VLCFA lowering effect in patients with X-ALD, a follow-up trial will be planned to determine if bezafibrate can delay or halt progression of the disease.

## 10.5 Compensation for injury

The sponsor/investigator has a liability insurance which is in accordance with article 7, subsection 6 of the WMO (please refer to the appendix with the patient information).

The sponsor (also) has an insurance which is in accordance with the legal requirements in the Netherlands (Article 7 WMO and the Measure regarding Compulsory Insurance for Clinical Research in Humans of 23th June 2003). This insurance provides cover for damage to research subjects through injury or death caused by the study.

1. € 450.000,-- (i.e. four hundred and fifty thousand Euro) for death or injury for each subject  who participates in the Research;
2. € 3.500.000,-- (i.e. three million five hundred thousand Euro) for death or injury for all subjects who participate in the Research;
3. € 5.000.000,-- (i.e. five million Euro) for the total damage incurred by the organisation for all damage disclosed by scientific research for the Sponsor as 'verrichter' in the meaning of said Act in each year of insurance coverage.

The insurance applies to the damage that becomes apparent during the study or within 4 years after the end of the study.

### 10.6 Incentives (if applicable)

Participants will be reimbursed for travel expenses. There are no other financial incentives to encourage participation.

# 11. ADMINISTRATIVE ASPECTS AND PUBLICATION

## 11.1 Handling and storage of data and documents

Since there is no randomization or blinding the data are stored uncoded. Data from the visits (side effects, physical examination) will be stored on the CRF. Data from routine clinical chemical analysis is stored in hospital data management system (Zorgdesktop), and will be stored for analysis on the CRF. All very long chain fatty acid measurements (from the laboratory for Genetic Metabolic Diseases) will be stored in the databases of that laboratory, already used for storage of test results for routine diagnostic tests, and will also be stored for analysis on the CRF. These databases are secure and well protected.
Only the investigators have access to this data. Plasma, leukocytes and erythrocytes isolated drom blood samples collected during the trial will be stored for possible future use (in anonymised form), if patients give their consent, otherwise the material will be destroyed after completion of the trial.

## 11.2 Amendments

A 'substantial amendment' is defined as an amendment to the terms of the METC application, or to the protocol or any other supporting documentation, that is likely to affect to a significant degree:

- the safety or physical or mental integrity of the subjects of the trial;
- the scientific value of the trial;
- the conduct or management of the trial; or
- the quality or safety of any intervention used in the trial.

All substantial amendments will be notified to the METC and to the competent authority.
Non-substantial amendments will not be notified to the accredited METC and the competent authority, but will be recorded and filed by the sponsor.

## 11.3 Annual progress report

The sponsor/investigator will submit a summary of the progress of the trial to the accredited METC once a year. Information will be provided on the date of inclusion of the first subject, numbers of subjects included and numbers of subjects that have completed the trial, serious adverse events/ serious adverse reactions, other problems, and amendments.

## 11.4 End of study report

The sponsor will notify the accredited METC and the competent authority of the end of the study within a period of 90 days. The end of the study is defined as the last patient's last visit.

In case the study is ended prematurely, the sponsor will notify the accredited METC and the competent authority within 15 days, including the reasons for the premature termination.

Within one year after the end of the study, the investigator/sponsor will submit a final study report with the results of the study, including any publications/abstracts of the study, to the accredited METC and the Competent Authority.

### 11.5 Public disclosure and publication policy

This is a non-commercial study. There are no limitations on disclosure and publication of the study results. The trial will be registered in http://www.ntr.nl and http://www.iscrtn.org

## 12. REFERENCES


1. Moser HW. Adrenoleukodystrophy: phenotype, genetics, pathogenesis and therapy. Brain 1997; 120 ( Pt 8):1485-1508.

2. Bezman L, Moser AB, Raymond GV et al. Adrenoleukodystrophy: incidence, new mutation rate, and results of extended family screening. Ann Neurol 2001; 49(4):512-517.

3. van Geel BM, Bezman L, Loes DJ, Moser HW, Raymond GV. Evolution of phenotypes in adult male patients with X-linked adrenoleukodystrophy. Ann Neurol 2001; 49(2):186-194.

4. Peters C, Charnas LR, Tan Y et al. Cerebral X-linked adrenoleukodystrophy: the international hematopoietic cell transplantation experience from 1982 to 1999. Blood 2004; 104(3):881-888.

5. Wanders RJ, van Roermund CW, van Wijland MJ et al. Direct demonstration that the deficient oxidation of very long chain fatty acids in X-linked adrenoleukodystrophy is due to an impaired ability of peroxisomes to activate very long chain fatty acids. Biochem Biophys Res Commun 1988; 153(2):618-624.

6. Ho JK, Moser H, Kishimoto Y, Hamilton JA. Interactions of a very long chain fatty acid with model membranes and serum albumin. Implications for the pathogenesis of adrenoleukodystrophy. J Clin Invest 1995; 96(3):1455-1463.

7. Kemp S, Pujol A, Waterham HR et al. ABCD1 mutations and the X-linked adrenoleukodystrophy mutation database: role in diagnosis and clinical correlations. Hum Mutat 2001; 18(6):499-515.

8. Aubourg P, Adamsbaum C, Lavallard-Rousseau MC et al. A two-year trial of oleic and erucic acids ("Lorenzo's oil") as treatment for adrenomyeloneuropathy. N Engl J Med 1993; 329(11):745-752.

9. van Geel BM, Assies J, Haverkort EB et al. Progression of abnormalities in adrenomyeloneuropathy and neurologically asymptomatic X-linked adrenoleukodystrophy despite treatment with "Lorenzo's oil". J Neurol Neurosurg Psychiatry 1999; 67(3):290-299.

10. Singh I, Khan M, Key L, Pai S. Lovastatin for X-linked adrenoleukodystrophy. N Engl J Med 1998; 339(10):702-703.

11. Sanchez RM, Vazquez M, Alegret M et al. Cytosolic lipogenic enzymes: effect of fibric acid derivatives in vitro. Life Sci 1993; 52(2):213-222.

12. Sanchez RM, Vinals M, Alegret M et al. Fibrates modify rat hepatic fatty acid chain elongation and desaturation in vitro. Biochem Pharmacol 1993; 46(10):1791-1796.

13. Wierzbicki AS. Fibrates in the treatment of cardiovascular risk and atherogenic dyslipidaemia. Curr Opin Cardiol 2009; 24(4):372-379.

## 13. Tables and Figures

Table 1

| | |
|---|---|
| Laboratorium voor Klinische Chemie | Na, K, creatinine, ureum, ASAT, ALAT, G-GT, AF, CK, cholesterol (LDL-, HDL-, totaal), triglyceriden, hemoglobine/Ht, leukocyten en differentiatie, thrombocyten, extra plasma stored at -80 C (heparine, EDTA) |
| Laboratorium voor Genetische Metabole Ziekten | VLCFA (in plasma, leukocyten, erythrocyten), C26:0-lyso-phosphatidylcholine, extra plasma stored at -80 C (heparine, EDTA) |

For each visit: 2 x 7 ml EDTA en 2 x 7 ml heparin blood, and 3 x bloodspot on standard filtration paper for the C26:0-lyso-PC levels.

Figure 1

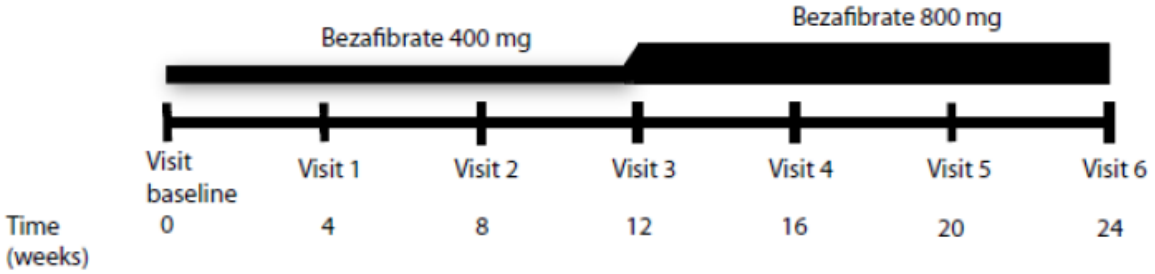

Supplement: Protocol S1 — Trial Protocol. (PDF) [file pone.0041013.s002.pdf]
